# Electricity up to Date
*"Electricity up to Date: Light, Power, and Traction." By John B. Verity, M.Inst.C.E. (London: Frederick Warne and Co.) Price, 1s. 6d.


**Published:** 1892-01-16

**Authors:** 


### * Electricity up to Date.

Mr. Verity has performed a useful public service by the publication of this monograph. It is clear, terse, practical, and complete. It contains chapters on the different modes of producing electricity, electricity as an illuminant, the storage of electricity, the wiring of houses, private installations, the public supply of electricity, Board of Trade regulations, electrical engineering as a calling, and many other points of interest. There is an excellent glossary and index, besides a number of illustrations in explanation of the text. Now that the electric light is on the eve of being introduced into most middle-class houses, a handy book like this was greatly wanted. We can recommend it to the heads of households, and should hope that it may speedily attain a large circulation, because the more members of the public become enlightened on the subject of electricity the better it will be for them and the community at large. Handy, lucid, and practical, this book should find its way into the hands of every enlightened member of the community. We ought to add that, amongst many other useful features, the book contains a map showing the areas allotted to the various electric supply companies in London, together with an account of the present position occupied by these companies and the work they are doing.

* "Electricity up to Date: Light, Power, and Traction." By John B. Verity, M.Inst.C.E. (London: Frederick Warne and Co.) Price, 1s. 6d.

